# Electrocardiogram and echocardiography findings and the outcomes of patients with myocardial infarction: Retrospective study in tertiary care hospitals in Northwest Ethiopia

**DOI:** 10.1371/journal.pone.0288698

**Published:** 2023-08-04

**Authors:** Wondale Tsega, Worku Awoke, Ashenafi Kibret Sendekie, Ephrem Mebratu Dagnew, Habtamu Bayih

**Affiliations:** 1 Department of Internal Medicine, College of Medicine and Health Sciences, Bahir Dar University, Bahir Dar, Ethiopia; 2 Department of Epidemiology, School of Public Health, College of Medicine and Health Sciences, Bahir Dar University, Bahir Dar, Ethiopia; 3 Department of Clinical pharmacy, School of Pharmacy, College of Medicine and Health Sciences, University of Gondar, Gondar, Ethiopia; 4 Depatment of Pharmacy, College of Medicine and Health Sciences, Debre Markos University, Debre Markos, Ethiopia; The University of Mississippi Medical Center, UNITED STATES

## Abstract

**Background:**

Myocardial infarction (MI) is diagnosed when there is a rise in cardiac biomarkers along with supportive evidence in the form of typical symptoms, suggestive electrocardiographic (ECG) changes, or imaging evidence of a new loss of viable myocardium or a new regional wall motion abnormality. The data regarding the use of ECG and echocardiography (Echo) findings and their impact on mortality are still lacking in Ethiopia. This study assessed the utilization of ECG and Echo findings and outcomes of patients with MI in tertiary care hospitals in Northwest Ethiopia.

**Methods:**

A retrospective chart review was conducted on patients with MI who were admitted to the adult intensive care units (ICUs) of two selected hospitals between January 2018 and July 30, 2021. Data was entered and analyzed using the SPSS 25 software. Logistic regression analysis was used to assess the association between in-hospital mortality and other variables. A P-value < 0.05 was considered significant.

**Results:**

Among the 203 participants, 67.5% were male, and the mean age of the participants was 59 (13.8). Around two-thirds (66.5%) of patients had STEMI and a regional all-motion abnormality. More than half (54.1%) of the cases were in the anteroapical region. For MI, there was a 23.2% inconsistency between ECG and Echo findings. The rate of in-hospital mortality for patients with MI was 23%. Pulmonary hypertension [AOR = 7.8, 95% CI: 1.72–34.93], inferobasal regional wall motion abnormality [AOR = 7.9, 95% CI: 1.340–46.093], Killip’s classes III and IV [AOR = 2.7, 95% CI: 1.103–6.314], infection [AOR = 3.2, 95% CI: 1.108–10.65], and ischemic stroke [AOR = 1.9, 95% CI: 1.091–5.222] were significantly associated with in-hospital mortality compared with their counterparts.

**Conclusions:**

The mortality of patients with MI in this study was higher than in other reports. Killip’s class, pulmonary hypertension, infection, ischemic stroke, and inferobasal regional wall motion abnormalities were significantly associated with the in-hospital mortality of the patients with MI. There was a higher degree of inconsistency between ECG and Echo findings. The treatment of patients with MI should be tailored to their specific risk factors and causes.

## Introduction

Myocardial infarction (MI) is defined pathologically as myocardial cell death due to prolonged ischemia. Criteria are met when there is a rise in cardiac biomarkers along with supportive evidence in the form of typical symptoms, suggestive ECG changes, or imaging evidence of a new loss of viable myocardium or a new regional wall motion abnormality [1]. The clinical presentation of myocardial ischemia is most often acute chest discomfort. The gold standard diagnostic modality for coronary artery disease is a coronary angiogram, but in patients with MI, cardiac biomarkers, ECG, and Echo have their own roles. Serial serum biomarkers (troponin T and I) are essential for confirming the diagnosis of infarction.

An ECG is a visual representation of the heart’s electrical activity. It amplifies and records the signals after sensing them using metal electrodes placed to the extremities and chest wall [2]. The atria’s activation causes the P wave, the PR interval is the length of the atrio-ventricular conduction, the two ventricles’ activation causes the QRS complex, and the ventricular recovery is reflected in the ST-T wave [3].

Echo uses high-frequency sound waves (ultrasound) to penetrate the body, reflect on relevant structures, and generate an image [4]. Regional wall motion should be assessed on multiple image views at the parasternal long-axis and short-axis views and the apical four chamber, two chamber, and three chamber views [5].

Coronary artery disease is a severe problem in Ethiopia, as demonstrated by its early onset and more widespread involvement of the coronary vasculature. It has been demonstrated that coronary intervention without on-site cardiac surgery is safe [6]. Acute MI continues to be a significant public health problem in industrialized countries and an increasingly significant problem in developing countries. The data regarding the ECG and Echo comparison and their use are still lacking in Ethiopia.

There are limited studies on the assessment of ECG and Echo findings and their impact on the outcome of patients with MI. The use of Echo in acute MI is less common even in cases of normal ECG findings, so this study will give direction to use bedside Echo as routine in acute coronary syndrome patients’ approaches. The study findings will contribute to the development of knowledge about ECG and Echo findings and their effect on the outcome, and they will serve as a reference for those who want to study related research topics. The findings of this study may also help in influencing the development of appropriate investigation and intervention programme for the management of MI. As a result, this study assessed the ECG and Echo findings and outcomes of patients with MI in tertiary care hospitals in Northwest Ethiopia.

## Methods and materials

### Study design and setting

A hospital-based retrospective cross-sectional study was conducted at Tibebe Gihon Comprehensive Specialized Hospital (TGCSH) and Felege Hiwot Comprehensive Specialized Referral Hospital (FHCSH) on patients with MI who were admitted between January 2018 and July 30, 2021. The hospitals were selected randomly from other hospitals located in Bahir Dar, Northwest of Addis Ababa. Both TGSH and FHRH have inpatient and outpatient departments, which have provided service for patients from the northwest part of Ethiopia. These hospitals have provided healthcare services for over 7 million people in their total catchment areas.

### Study participants, eligibility criteria, and sampling procedures

All patients with MI who were admitted to the TGCSH and the FHCSH were in the study population. Patients with MI who were admitted to the selected hospitals during the study period and patients with troponin, ECG, and/or Echo who fulfilled the fourth universal definition of MI were included in the study. The sample size was determined by using the following assumptions: Based on the second objective of the Echo examination, regional wall motion abnormalities were found in 52.8% of patients in the CAD group [7], with a 95% confidence level of 1.96 and a 5% acceptable margin of error (400 patients were admitted to the ICU of two hospitals from January 2018 to July 2021 with a diagnosis of MI using EPI Info version 7, and considering 10% contingency, the final sample size was 216). Simple random sampling was used to approach the participants using their medical registration number from the ICU logbook record.

### Definition of some terms

#### Typical chest pain

in acute MI, it has the following characteristics: substernal and often radiates up to the neck, shoulder, and jaw, as well as down the left arm, and is usually described as a substernal pressure sensation that can also be described as squeezing, aching, burning, or even sharp.

#### Global LV systolic function

An Echo assessment was used to determine global LV systolic function, in this case, left ventricular ejection fraction (LVEF). Mildly depressed (41 to 51 for men and 41 to 53 for women), moderately depressed (30 to 40), and severely depressed (< 30) are the classifications.

#### Pulmonary hypertension

In this study, pulmonary hypertension was defined as a pulmonary artery systolic pressure (PASP) of more than 36 mmHg as determined by Echo. Normal PASP (PASP 35 mmHg), mild (PASP 36–45 mmHg), moderate (PASP 46–60 mmHg), and severe PH (PASP > 60 mmHg) are the four levels of PASP [8].

#### Diastolic dysfunction

The finding and grade of diastolic dysfunction in this study are based on the echocardiographer’s report, which is rated from mild (grade I) to severe (grade II) (grade III).

### Fourth universal definition of myocardial infarction

The term acute myocardial infarction should be used when there is acute myocardial injury with clinical evidence of acute myocardial ischemia and with detection of a rise and/or fall of cTn values with at least 1 value above the 99th percentile URL and at least 1 of the following [1]:

➢ Symptoms of myocardial ischemia;
➢ New ischemic ECG changes;
➢ Development of pathological Q waves;
➢ Imaging evidence of a new loss of viable myocardium or a new regional wall motion abnormality in a pattern consistent with an ischemic etiology
➢ Identification of a coronary thrombus by angiography.

#### Data collection procedures and quality control measures

A semi-structured data collection format was used to collect the data. The data collection tool was prepared in English after reviewing earlier studies. The data was collected by reviewing patients’ medical records. We gathered the patient’s sociodemographic, medication, and clinical information, as well as ECG and Echo results.

The ECG changes to say the patient had MI are: ST-elevation (a new ST-elevation at the J-point in two contiguous leads with a cut-point of ≥ 1 mm in all leads except leads V2-V3, which have the following cut points: ≥ 2 mm in men ≥ 40 years old; ≥ 2.5 mm in men < 40 years old; or ≥ 1.5 mm in women of any age and ST-depression and T-wave changes (new horizontal or down-sloping ST-depression of ≥ 0.5 mm in two consecutive leads and/or T inversion of more than > 1 mm in two consecutive leads with a prominent R wave or R/S ratio > 1. ECG changes associated with prior MI (in the absence of LVH and BBB) include any Q wave in leads V2–V3 > 0.02s and a QS complex in leads V2–V3 or Q waves ≥ 0.03 s and ≥ 1 mm deep in any two contiguous leads (I, aVL; V1-V6; II, III, aVF), or a QS complex. * R wave > 0.04 s in V1–V2 and R/S >1 with a concordant positive T wave in the absence of a conduction defect [1]. The anatomic location of MI changes by ECG is based on the affected specific lead of a 12-lead ECG: anterior wall (V1-V6 and I and aVL), anterio-septal (V1-V3), anterio-lateral (V4-V6), inferior wall and RV (II, III, aVF, and V3R and V4R), posterior wall (V7-V9 and depression on V1-V2), inferior-posterior (V7-V9 and II, III and aVF), and multiple (two or more of the listed above). The Echo results are based on the report of the Echo-cardiographer.

To ensure the quality of the data collection tool, a pretest was conducted on patient medical records using 10% of the total sample size before the actual data collection period. In addition, to maintain the data’s uniformity, the three data collectors—internal medicine residents—were trained about the data collection tool and the ethical issues. Furthermore, the principal investigator supervised the entire data collection procedures, the data collection, and incomplete checklists were cleaned and checked for quality prior to data entry. The missing data was handled as follows: if the record did not contain an ECG, it was excluded from data collection; if the patient’s outcome was missing, it was included after verification in the ICU and ward logbooks.

#### Outcome measures

The primary outcome of this study was ECG and Echo findings (Table 1).

**Table 1:** Electrocardiographic changes associated with myocardial infarction.

ECG changes (in the absence of LVH and BBB)	Description
ST-elevation	A new ST-elevation at the J-point in two contiguous leads with a cut-point of ≥ 1 mm in all leads except leads V2-V3, which have the following cut points: ≥ 2 mm in men ≥ 40 years old; ≥ 2.5 mm in men < 40 years old; or ≥ 1.5 mm in women of any age
ST-depression and T-wave changes	New horizontal or down sloping ST-depression of ≥ 0.5 mm in two consecutive leads and/or T inversion of more than > 1 mm in two consecutive leads with a prominent R wave or R/S ratio > 1
Prior MI	Any Q wave in leads V2–V3 > 0.02s and a QS complex in leads V2–V3 or Q waves ≥ 0.03 s and ≥ 1 mm deep in any two contiguous leads (I, aVL; V1-V6; II, III, aVF), or QS complex. * R wave > 0.04 s in V1–V2 and R/S >1 with a concordant positive T wave in the absence of a conduction defect

### Data entry and analysis

The data was entered and analyzed using SPSS version 25. The characteristics of the study population were described by descriptive statistics. The normal distribution of the data was examined using Q-Q plot and histogram. The characteristics of the study population were described by descriptive statistics. Binary logistic regression is used to determine whether ECG or Echo findings or other factors influence patient outcomes (in-hospital death). In the univariable analysis, variables with a p value of 0.2 were considered for the multivariable analysis. A p value of less than 0.05 at a 95% confidence level was considered statistically significant.

### Ethical considerations

Ethical clearance was obtained from the Bahir Dar University Ethics Review Committee with a reference number of 0035/21. A support letter was sent to Tibebe Ghion Specialized Hospital and Felege Hiwot Referral Hospital. The institutional review boards or ethics committees waived the requirement for written informed consent. Identifiers such as names were not used in collecting the data. Confidentiality was maintained by keeping the data collection forms locked in a secure cabinet, and the electronic data file was kept securely on a password-protected computer. All methods were carried out in accordance with relevant guidelines and regulations.

## Results

### Sociodemographic and clinical characteristics of the participants

Out of 216 patients admitted with the diagnosis of MI, 203 (94%) who fulfill the fourth definition of MI were included in this study. Around two-thirds of the participants (137, or 67.5%) were male. The mean age of the participants was 59±13.8 years. About 80.3% of patients presented with typical chest pain for MI. In addition to chest pain, patients reported shortness of breath (27.1%), diaphoresis (25.6%), and epigastric burning pain (16.7%). In terms of Killip’s class, 47.8% of the patients were classified as Killip’s class I, while 20.2% were classified as Killip’s class III (**Table 2).**

**Table 2:** Sociodemographic and clinical parameters of patients with MI at TGSH and FHRH between January 2018 and July 2021 (N = 203).

Variables	Categories	Frequency	Percent (%)	Mean (SD)
Age categories	< 50 years	63	31.0	59±13.8
	50–59 years	28	13.8	
	60–69 years	64	31.5	
	70–79 years	32	15.8	
	80 years and above	16	7.9	
SEX	Male	137	67.5	
	Female	66	32.5	
Chest Pain	Typical	163	80.3	
	Atypical	23	11.3	
	No chest pain	17	8.4	
Killip’s Class	Class I	97	47.8	
	Class II	29	14.3	
	Class III	41	20.2	
	Class IV	31	15.3	
	Not documented	5	2.5	

### Comorbidities and risk factors

Out of 203 patients admitted with the diagnosis of MI, 47.3% had hypertension, 25.1% had diabetes mellitus (type II), 7.4% had retroviral infection (RVI), and 7.4% had dyslipidemia. Only 39 (40.6%) of MI patients with hypertension were receiving treatment. Of the patients with diabetes, three-fourths (75%) were on treatment. All of the RVI patients were on HAART (**Table 3**).

**Table 3:** Comorbidities and risk factors of patients with MI at TGSH and FHRH.

Variables	Categories	Frequency	Percent (%)
Diabetes mellitus	Yes	51	25.1
	No	152	74.9
Hypertension	Yes	96	47.3
	No	107	52.7
Ischemic stroke	Yes	31	15.3
	No	172	84.7
Pulmonary hypertension	Yes	25	12.3
	No	178	87.7
Smoking	Current Smoker	8	3.9
	Previous Smoker	2	1.0
	No Smoking	193	95.1
Alcoholism	Yes	10	4.9
	No	193	95.1
Dyslipidemia	Yes	15	7.4
	No	114	56.2
	Lipid profile not done	74	36.5
RVI status	Yes	15	7.4
	No	188	92.6
Other infection	Yes	38	18.7
	No	165	81.3
Ejection fraction	< 40	60	29.6
	>40	143	70.4

### Electrocardiogram and echocardiography findings

#### Electrocardiograph findings

**Fig 1:**
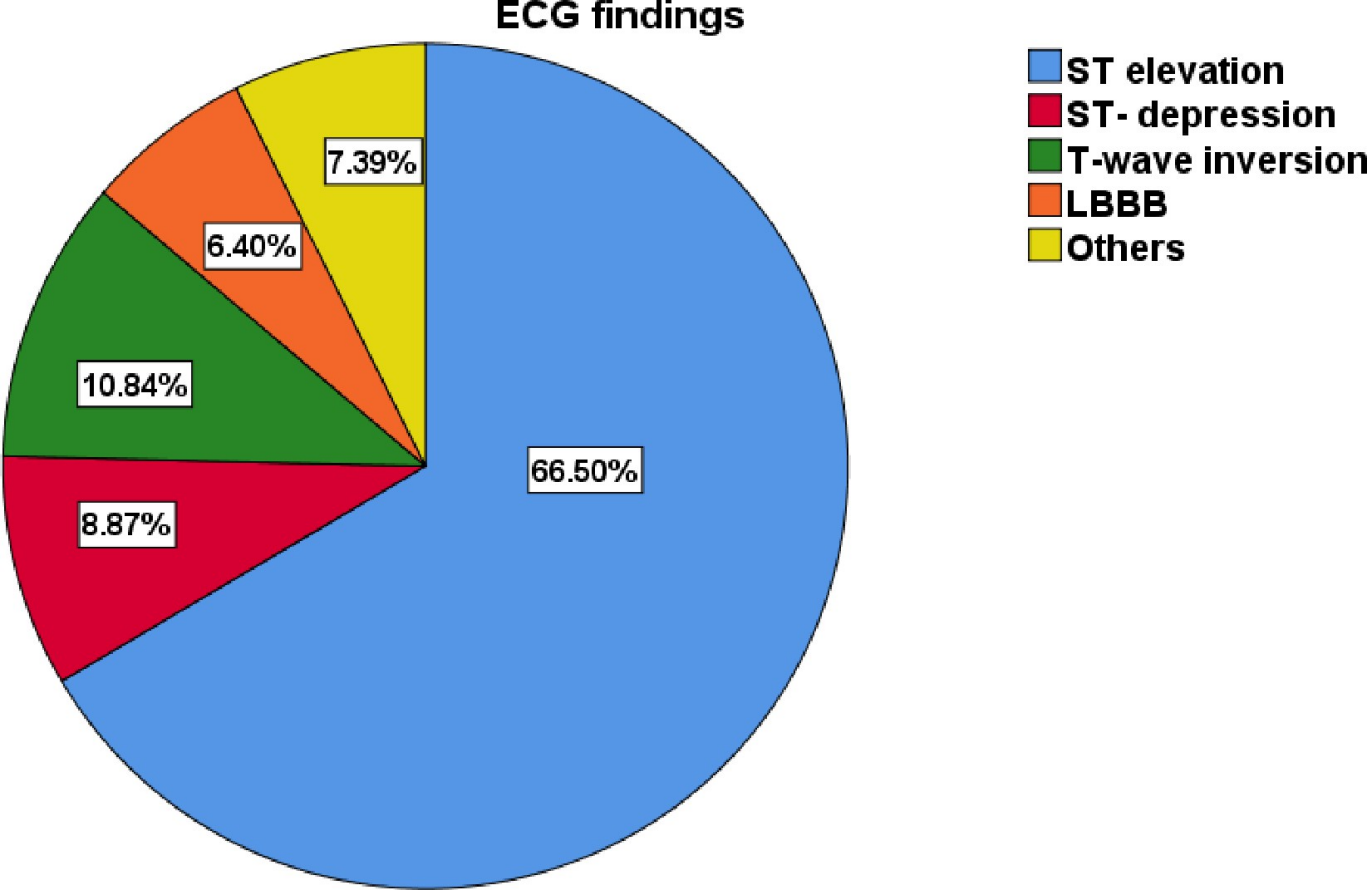
Common ECG findings of patients with MI at TGSH and FHRH between January 218 and August 2021 (N = 203).

In this study, around two-thirds (135, 66.5%) of patients had STEMI, 42 (20.7%) had NSTEMI, and 26 (12.8%) had no ECG changes. About 66.5% of the patients had ST-elevation, 10.8% had T-wave inversion, 8.9% had ST-depression, and 6.4% had LBBB (**Fig 1**). The ECG anatomic location of the infract anterior wall accounts for 32% and the antero-septal area accounts for 25.1% (**Table 4**).

**Table 4:** Location infract by ECG of patients with MI patients.

		Frequency	Percent
ECG location infract	Anterior wall	65	32.0
	Antero-septal	51	25.1
	Antero-lateral	26	12.8
	Inferior wall	23	11.3
	Multiple	8	3.9
	No specific location	30	14.8

### Echocardiography findings

Regarding the Echo results of the study, only 146 (71.9%) patients had Echo. The majority of those 122 (83.5%) had MI findings, while 24 (11.8%) had no findings suggestive of MI. The systolic functions of those participants showed that 60 (41.1%) had a mildly reduced ejection fraction and 46 (31.5%) had a normal ejection fraction. 112 (76.7%) patients had no diastolic dysfunction, and 31 (21.2%) of them had grade I diastolic dysfunction. 82.9% (121) of patients had normal pulmonary arterial pressure, 97.3% (142) had normal TAPSE, and 78.8% (145) had a hypokinesis wall motion score index. Regarding MI complications detected by Echo, 9 patients (6.2%) had LV thrombus, 18 patients (12.3%) had mild mitral regurgitation, and 115 patients (78.8%) had no complications detected. Regarding regions, infract anteroapical accounts for 54.1% (79) of patients, and infero-basal accounts for 14.4% (21) (**Table 5**).

**Table 5:** Echo localization of segmental wall motion abnormalities of patients with MI.

Wall motion abnormalities		Frequency	Percent
**Wall motion segmental location**	Anterior wall (Base/Mid)	7	3.4
	Anterio-septal (Base/Mid)	25	12.3
	Anterio-lateral (Base/Mid)	6	3.0
	Septal only (Base/Mid)	9	4.4
	Inferior wall (Base/Mid)	5	2.5
	Apex (Anterior/Septal/Lateral/Inferior)	25	12.3
	Inferio-lateral (Base/Mid) or Posterio-lateral	10	4.9
	Multiple segment	31	15.3
	No RWMA	28	13.8
**Regional wall motion abnormality location**	Anteroapical	79	38.9
	Inferobasal	21	10.3
	Lateral or free wall	6	3
	Multiple segments	12	5.9
	No RWMA	28	13.8

### Inconsistency between ECG and ECHO findings

**Fig 2:**
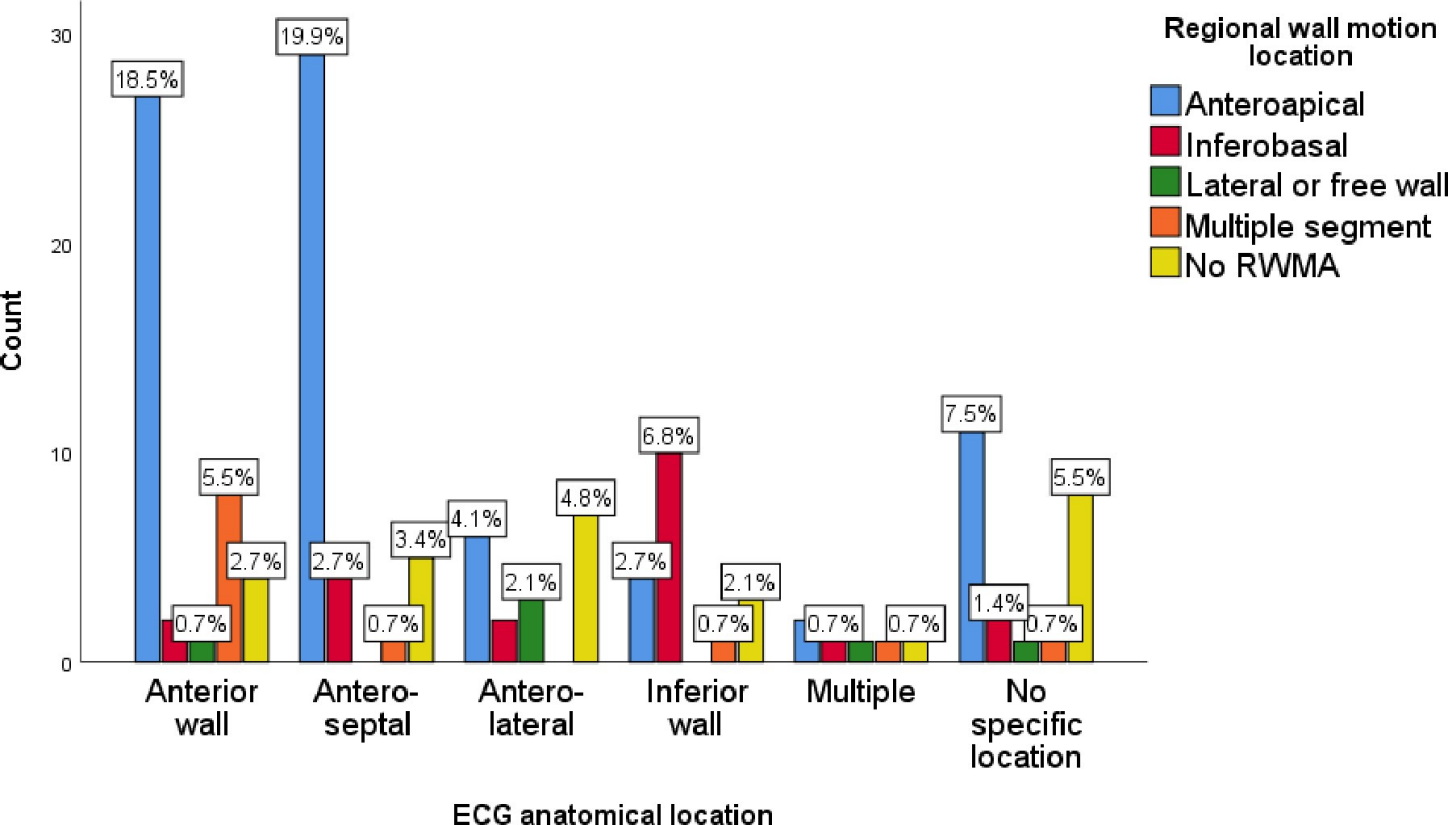
Echo regional wall motion location and ECG location of infarction in patients with MI at TGSH and FHRH between January 2018 and July 2021(N = 203).

From those patients who had both an ECG and an Echo (146), 19 (13%) patients had an ECG suggestive of MI but no RWMA on Echo, and 15 (10.2%) patients had no suggestive ECG finding for MI but an Echo finding suggestive of MI (there was RWMA). Overall, there was a 23.2% inconsistency between ECG and Echo findings in suggesting MI. There was consistency in showing the regional location of the infraction in 78 (53.4%) of patients who had both an ECG and an Echo. We analyzed the association between the ECG location of infract and Echo regional wall motion abnormalities with chi-square, and the finding suggested that there is a significant difference with p < 0.001. In 25 (17.1%) of patients with apical hypokinesis detected by Echo, 32% had antero-septal MI and 24% had anterior wall MI on ECG (56% have both antero-septal and anterior wall MI on ECG) (**Fig 2**).

### In hospital outcomes

**Fig 3:**
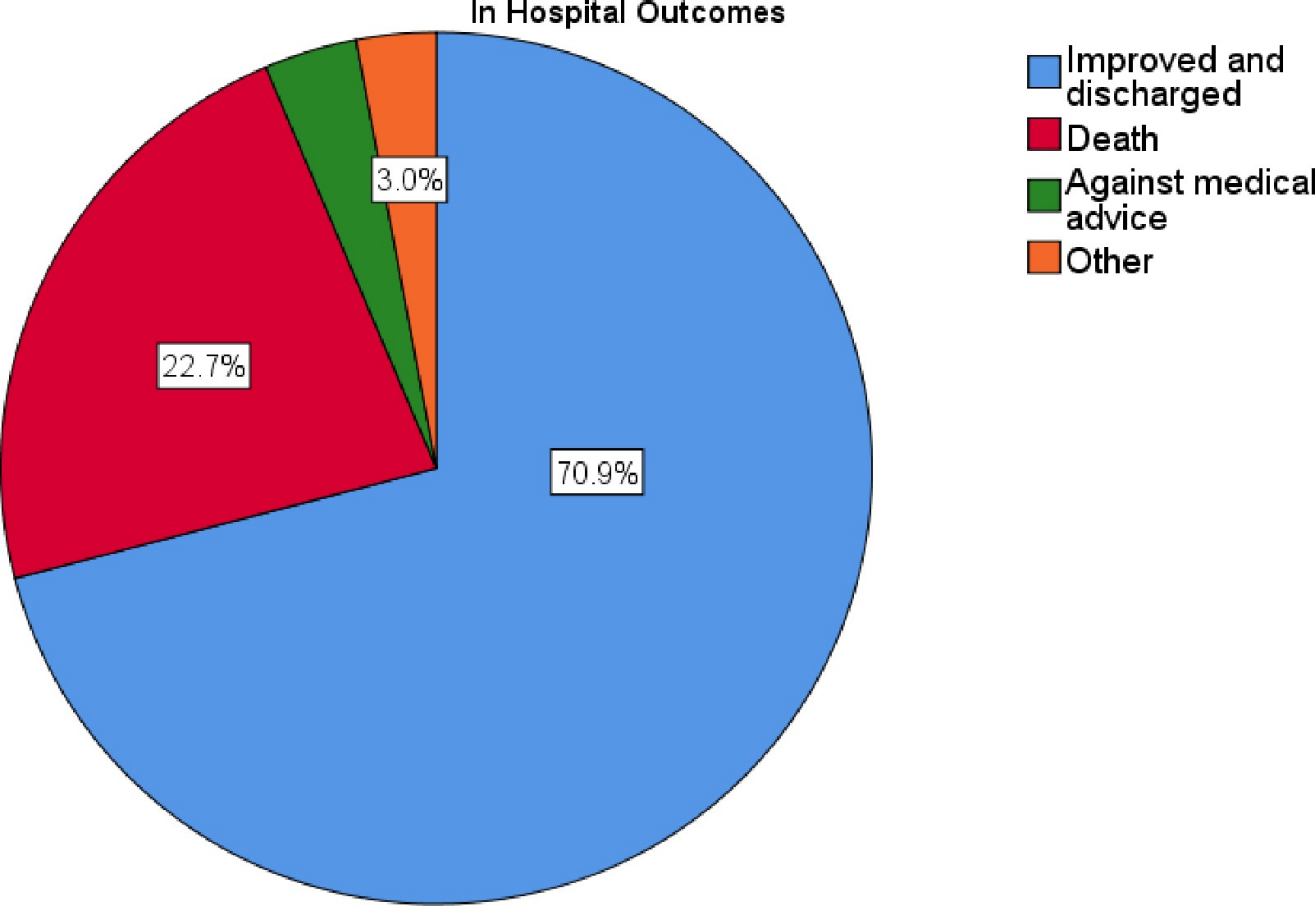
In hospital outcome of patients with MI at TGSH and FHRH between January 2018 and July 2021(N = 203).

Out of 203 admitted patients with MI, about 46 (22.7%) died, and 144 (70.9%) were discharged with improvements (**Fig 3**).

When the death rate in this study was compared to each ECG finding of MI, six patients with LBBB died (50%), while 22.2% died from STEMI. According to the ECG location of the infract, 32.2% (20 out of 62) of patients with anterior wall MI died.

When the death rate for each Echo finding was compared, four out of eleven patients (36.3%) died in patients with an EF of < 30%. It was higher in those with infero-basal MI (28.5%) based on the location of the regional wall motion abnormality. In terms of location of segmental wall motion abnormality, more than 40% of those with inferolateral or posterior-lateral abnormalities died; 5 died out of 7 patients with severe pulmonary hypertension (71.4%), and 4 died out of 10 patients with moderate pulmonary hypertension (40%).

### Predictors of in-hospital death outcomes

When we look at the in hospital all-cause mortality, 46 (24.2%) patients died out of 190, patients and the other 13 patients were unknown whether they died or not.

Using multivariable logistic regression analysis, findings that had a statistically significant association with in-hospital death were pulmonary hypertension [AOR = 7.859, 95% CI (1.72–34.93); p = 0.008], inferobasal regional wall motion abnormality [AOR = 7.859, 95% CI (1.340–46.093); p = 0.022], Killip’s class III and IV [AOR = 2.728, 95% CI (1.103–6.314); p < 0 .001], presence of infection [AOR = 3.236, 95% CI (1.108–10.65); p = 0.032], and the presence of ischemic stroke [AOR = 1.871, 95% CI (1.091–5.222); p = 0.023] compared with their counterparts (**Table 6).**

**Table 6:** Multivariate logistic regression analyses of factors affecting in hospital mortality of patients with myocardial infarction.

Variable	Categories	In Hospital death		AOR	95% Confidence interval for AOR		P-Value
		Yes	No				
Age	≥ 50 years	15	48	1.723	0.293	10.130	0.547
	< 50 years	31	106	1.00			
Sex	Male	37	98	2.079	0.485	12.673	0.275
	Female	9	46	1.00			
Hypertension	Yes	20	63	1.091	0.257	1.918	0.350
	No	26	81	1.00			
RVI	Yes	4	11	1.098	0.051	3.308	0.053
	No	42	133	1.00			
Ischemic stroke	Yes	8	22	1.871	1.091	5.222	0.023*
	No	38	122	1.00			
Killip’s class grouped	III & IV	25	45	2.728	1.103	6.314	< 0.001*
	I & II	21	99	1.00			
Infection	Yes	16	20	3.236	1.108	10.652	0.032*
	No	30	124	1.00			
Pulmonary Hypertension	Yes	14	8	7.755	1.722	34.930	0.008*
	No	32	136	1.00			
Regional wall motion location	Infero-basal	17	10	7.859	1.340	46.093	0.022*
	Anteroapical	29	134	1.00			
Ejection fraction	EF < 40	12	35	1.696	0.336	8.561	0.523
	EF > 40	34	109	1.00			
Diastolic Dysfunction	Yes	10	40	0.454	0.084	2.458	0.359
	No	36	104	1.00			

*Indicates significant variables at p< 0.05

## Discussion

The current study’s findings from ECG and Echo showed that pulmonary hypertension and infero-basal regional wall motion abnormalities have a significant association with the mortality of the patients. Killip’s class, infection, and ischemic stroke were associated with the in-hospital mortality of the patients with myocardial infarction.

The all-cause mortality rate of patients with MI in this study was closer to the study done at Saint Pitter Hospital and Jimma University Hospital in Ethiopia [9]. However, the in-hospital mortality in our setup is five times higher than the report from the ACTION (Acute Coronary Treatment and Intervention Outcomes Network) Registry–the WTG (Get with the Guidelines) [10]. While some factors have extensive global effects (e.g., hypertension), but in low-income and middle-income countries, household air pollution, poor diet, low education, and low grip strength had stronger effects on cardiovascular disease or mortality than in high-income countries [11]. Additionally, most hospitals high income countries have a cardiac center with revascularization procedures such as percutaneous coronary intervention (PCI) and fibrinolytic therapy, but our hospital does not have this setup. So, the current data supports the importance of timely reperfusion of jeopardized myocardium as the most effective way of restoring the balance between myocardial oxygen supply and demand [1].

In those patients having both Echo and ECG, there was a 23.2% inconsistency between ECG and echocardiography findings in locating an anatomic ischemic site and suggesting MI findings in one fourth of patients; this result is closer to the multi-centre study done by Kuch J et al, where 27.8% of the inconsistencies of ECG and Echo-2D evaluations were demonstrated [12]. In this study, all patients had ECG, but for one-fourth of them, an Echo was not done. Those patients with no Echo have higher mortality (49.1%), This might be because of patients died early before Echo was done. Patients had been sent to Echo after some days in the ICU. Using Echo early in the diagnosis of MI is not practiced, but early 2D Echo provides superior prognostic information concerning the risk of subsequent complications in patients with acute chest pain and a non-diagnostic ECG for ST-elevation-AMI [13]. In this study, one fifth of patients had NSTEMI (STD and TWI); those patients had sub-endocardial ischemia, and the distribution of STD in the 12 ECG leads did not correlate with the locations of wall motion abnormalities in Echo [2]. In contrast to the findings of the Poland study by Kuch J et al., more than half of the patients with apical hypokinesis that was detected by Echo also had both antero-septal and anterior wall MI. Different MI localizations were identified by ECG in 209 individuals (32%) with myocardial contractility abnormalities in the apical area of the heart who had been diagnosed by Echo-2D [12]. On a 12-lead ECG, there are no unique leads for localizing apical wall infarction.

When the death rate was compared to each ECG finding of MI in this study, half of patients with LBBB died. LBBB and RBBB in the setting of acute MI also portend a similar poor prognosis and should prompt consideration of urgent catheterization [3], and mortality is higher in patients with STEMI than NSTEMI. The death rate was higher in patients with anterior wall MI. When each Echo finding was compared to death, death was higher in those with EF < 30. A high risk of death was associated with the presence of a regional wall motion abnormality in those with inferobasal MI; this may be because patients have inferior wall MI has several complicating factors that increase mortality, including right ventricular infarction, hypotension, bradycardia, heart block, and cardiogenic shock [14], so in managing patients with inferior wall MI, we need to address those complications cautiously. Pulmonary hypertension detected by Echo had a statistically significant association [AOR = 7.755, 95% CI (1.72–34.93)] with all-cause mortality in MI patients; this is similar to the result found in the study by Marlieke et al, where incidentally elevated SPAP was independently associated with all-cause mortality [15]. This may be due to the fact that pulmonary hypertension affects the lung’s function of oxygen exchange and fastens mortality in already affected myocardial muscle.

In our study, patients having Killip classes IV and III were associated with increased mortality [AOR = 2.728, 95% CI (1.103–6.314) this is similar to a previous study where Killip classes III–IV were an independent predictor of in-hospital mortality [16], so the killip classification performed at the time of admission is a simple and useful clinical marker of a high risk of early and late adverse cardiovascular events [16]. Patients with any infection have an increased risk of MI and hospital death (P = 0.032), and in a previous study, infections complicating the course of patients with STEMI were uncommon but associated with markedly worse 90-day clinical outcomes [17]. Mechanisms for early identification of these high-risk patients as well as the design of strategies to reduce their risk of infection are warranted [17]. In this study with MI, those who have ischemic stroke (IS) are associated with increased mortality (P = 0.023). This result is in line with previous studies showing patients with ischemic stroke with a medical history of MI have an increased risk of severe stroke, in-hospital mortality, and complications [18]. The underlying mechanism of higher complications in patients with IS with MI remain unknown and need further exploration and tailored stroke treatment [18]. Although it was not statistically significant, older and female patients had higher mortality than younger and male patients, respectively. This finding is similar to the study done by Smilowitz NR [3]. Age is one of the most significant prognostic parameters of post-MI death, partially due to the high risk of vascular events after MI in elderly patients [4] and females who presented with atypical symptoms and arrived late at the hospital. As a result, women may experience symptoms of STEMI differently than men, requiring particular awareness and vigilance on the part of the interviewing physician [1].

In this study, two-thirds of the patients were male, and the mean age was 5913.8, which is closer to an earlier study [19]. Males are more affected than females; women have a delayed onset of MI compared to men, and it has been hypothesized that their premenopausal natural estrogen status has a protective impact. Female sex hormones have been associated with a less atherogenic lipid profile and a healthier fat distribution. Regarding lifestyle, the prevalence of smoking is highest in men, and women have healthier dietary habits [20]. MI and other ischemic heart diseases affect men in population subgroups that appear to be particularly affected, especially in South Asian countries, especially India and the Middle East [5]. More than three-fourths of patients presented with typical chest pain, which is in line with an Indian study in which 88% of patients presented with chest pain and 41% with breathlessness [21]. In another Indian study, chest pain was the most common presenting symptom [22].

In the current study, hypertension and diabetes mellitus are the two commonest risk factors, while RVI and dyslipidemia are seen in some patients. This is closer to the study done in Thanjavur, India, where type II diabetes mellitus was the most common modifiable risk factor [22]. This similarity might be because type 2 diabetes mellitus is increasing and is a powerful risk factor for IHD [5].

In this study, two-thirds of patients had STEMI, which is different from other studies done in the USA, in which STEMI constitutes 40% of all AMIs [23]; this may be due to socioeconomic differences, community awareness of the disease, early arrival to the hospital, and early screening for ischemic heart disease for those at risk in the USA. In this ECG study, AWMI accounts for 32.4 percent and is more common than IWMI, which accounts for 11.2 percent, which is similar to the Indian study. AWMI (51.27%) was more common than IWMI (46.19%) (14), since AWMI is more common than IWMI. Most of the patients in this study had a reduced ejection fraction. If a sufficient quantity of myocardium undergoes ischemic injury, LV pump function becomes depressed [5], and individuals with a drastically decreased ejection fraction have a greater death rate. Our findings emphasize the necessity of treating heart failure with low LVEF as soon as possible because angiotensin-converting enzyme inhibitors, b-blockers, and implantable cardioverter-defibrillators may significantly improve MI patients’ survival rates [4]. The ejection fraction of the participants was 26.6% with EF < 40% and 69% with EF < 51%, based on the location of the infract AWMI by ECG. 32.5% of those had EF 40%; based on Echo patients with AWMI, 42.8% had EF < 40%, which differs from the study done in south India, where there were more patients with LVEF < 40% in the AWMI group (64.4%) [22]. This finding might be because of the time difference in Echo during the hospital stay, and the method of ejection fraction estimation by the echocardiographer may be different in the two study areas.

In general, this study shows that there is a higher degree of inconsistency between ECG and Echo findings and that the treatment of patients with MI should be tailored to their specific risk factors. The study further highlights the necessity for real-time Echo, standardized ECG, and readily available ECG monitors in both emergency and intensive care units to ensure optimal patient care.

## Limitation and strength of this study

The current study has some limitations. It is a retrospective study with incomplete documentation. Some sensitive parts of medical history, like smoking, alcoholism, and tobacco chewing, were not completed and documented, which had an impact on the presentation of full and comprehensive participant characteristics in the study. Some sensitive parts of medical history, like smoking, alcoholism, and tobacco chewing, were not completed and documented, which had an impact on the presentation of full and comprehensive participant characteristics in the study. This may have an impact on the generalizability of the list of independent variables. In addition, the study design itself couldn’t determine causality or the long-term outcome. Furthermore, other clinical characteristics might be inconsistently recorded in the patient’s medical records. A relatively low proportion of patients were examined using Echo as compared with ECG, which may have had an effect on the comparative outcomes. Despite these limitations, the current study presents a comparative analysis of Echo and ECG findings in patients with MI, which is the first study of its kind in Ethiopia, especially in the study setting. It can add to the body of knowledge for patients, carers, and practitioners. The findings can also be used as a baseline for future studies. As a result, this study would welcome prospective research using a relatively larger sample.

## Conclusion

MI mortality is significantly higher than in other parts of the world. In around one-fourth of the MI patients, there were inconsistencies between the ECG and Echo in locating the site of the infraction. Killip’s class, pulmonary hypertension, infection, ischemic stroke, and inferobasal regional wall motion abnormalities were significantly associated with the in-hospital mortality of the patients with myocardial infarction. The treatment of patients with MI should be tailored to their specific risk factors and causes. Furthermore, at both emergency and intensive care units, real-time Echo, standardized ECG, and ECG monitors should be available at all times.

## Supporting information

S1 Checklist STROBE checklist. (DOC)

S2 Checklist *PLOS ONE* clinical studies checklist. (DOCX)

S1 File Minimal data set used in analyzing and generating the data. (SAV)

## Abbreviations

AMI: Acute myocardial infarction
AWMI: anterior wall myocardial infarction
CAD: coronary artery disease
ECG: electrocardiogram
Echo: echocardiography
EF: ejection fraction
IWMI: inferior wall myocardial infarction
LAD: left anterior descending
LBBB: left bundle branch block
MI: myocardial infarction
NSTEMI: Non-ST elevation myocardial infarction
RBBB: right bundle branch block
RCA: right coronary artery
RV: right ventricle
RVI: retroviral infection
RWMA: regional wall motion abnormality
STD: ST depression
STEMI: ST elevation myocardial infarction
TWI: T-wave inversion
