# Supplementary material for: Electrocardiogram and echocardiography findings and the outcomes of patients with myocardial infarction: Retrospective study in tertiary care hospitals in Northwest Ethiopia
Source: PLoS One. 2023 Aug 4;18(8):e0288698. doi: 10.1371/journal.pone.0288698 (PMC10403055; doi:10.1371/journal.pone.0288698)
Supplement: S2 Checklist — (DOCX) [file pone.0288698.s002.docx]

***PLOS ONE* Clinical Studies Checklist**

***PLOS ONE* manuscript number: PONE-D-23-07382**

| **Complete the following if your study involved human participants or human subjects’ data. These questions should be addressed for retrospective studies.** | | |
| --- | --- | --- |
| 1. | Did you obtain ethics approval for this study?   - If yes, please upload (file type “Other”) the original approval document you received from your ethics committee. If the original document is in another language, please also provide an English translation. - Yes, it was ethically approved - The approval letter is uploaded in the recommended file type. |  |
| 2. | If your study involved human participants, please report in the Methods section when participants were recruited to the study.   - The study period when the participants were recruited is reported in the study setting sections page 4, lines 22-30 and the inclusion criteria was also reported in page 4, lines 22-30. |  |
| 3. | If you are reporting a study of medical records or archived samples, please report in the Methods section the date range in which human subjects’ data/samples were collected and the date(s) when you conducted this study.   - N/A |  |
| 4. | Please specify in the Methods section whether authors had access to information that could identify individual participants during or after data collection.   - It is reported in the ethical approval and consent section in page 6 hat the data is sufficiently anonymized. |  |
| 5. | If you are reporting an observational study – i.e. cohort, case-control, and cross-sectional studies – we recommend that the work is reported as per the requirements of the STROBE guidelines, and that you provide a completed STROBE checklist as a Supporting Information file with your submission.   - STROBE checklist is Completed |  |
| 6. | Please ensure that the author list and Corresponding Author entered in Editorial Manager match the author list and Corresponding Author in your manuscript file.   - Ensured and we have listed and entered the authors and their affiliations correctly. |  |
